# Tuberculosis of Bones and Joints

**Published:** 1910-08

**Authors:** Theodore Toepel

**Affiliations:** Atlanta, Ga.


### TUBERCULOSIS OF BONES AND JOINTS.

BY THEODORE TOEPEL, M. D., ATLANTA, GA.

*Bone Tuberculosis.*—This may attack bone of every class, cartilaginous or membranous, and bony tissue of any type, cancellous or compact; or of any shape or location. It prefers cancellous tissue; and its choice of location is the newly-formed immature ossifying tissue in the ends of the long bones, near the epiphyseal line. In the short bones it locates centrally.

*Joint Tuberculosis.*—The disease of a joint generally begins is one of the bones composing the joint, although it may begin in the rynovial membrane. If beginning in the heaa of a long bone it may find its way through into the joint by destroying a part of the articular cartilage; or it may emerge at the margin of the epiphyseal line, and in those articulations whose epiphyseal line is within the capsule the disease is readily communicated to the joint.

The time allowed does not permit me to enter into a description of tuberculosis occurring in rynovial membrane, tubercular hydrops, tubercular fungous rynovitis by Bilroth and rynovitis hyperplastica granulosa by Hueter.

*Symptoms and Diagnosis.*—The classical signs of inflammations are considerably modified when the inflammatory process is of a tubercular nature, and especially if it be located in bone or joint.

Local heat upon the surface is not perceptibly increased.

Pain is dull and aching and worse at night. The night cries of children, moaning and restlessness and grinding of the teeth in sleep, while not pathognomonic, always call for a thorough examination for tubercular bone disease. Pain is not always felt at the point of the disease.

Tenderness is a more constant and reliable symptom than pain. A tubercular process in bone, if it approaches anywhere near the periosteum, produces a localized sensitive or tender spot, which can be found if searched for, and may be the only point of differentiation between a tubercular synovitis and an osteitis.

Swelling is a symptom in the majority of cases of joint tuberculosis. It is caused by thickening of the synovial membrane and fungus growths from its surface or by an effusion into the joint in diffuse synovitis, or by inflammatory exudate outside of the joint.

*Fluctuation* is present in hydrops and in intraarticular abcess, and pseudo-fluctuation is present when the joint is filled with fungus growths. he aspirating needle will differentiate.

*Atrophy of Muscle and Bone* is a symptom common to tuberculosis of both bones and joints. According to Vulpius the cause is tropho-neprotic, the irritation of the ends of the articular nerves reflect to the spinal centers, and from there upon the centers of the muscular nerves.

*Flexion with Loric Spasm of the Muscles* is present and usually an early symptom in all cases of active tubercular joint disease.

*Shortening and Displacements* are among the symptoms of joint tubercudosis in its later stages. Subluxations and malpositions are due to muscular contractions while the joint is in partial flexion, rotation or lateral deviation.

*Differential Diagnosis.*—Tuberculosis of bones and joints must be differentiated from rheumatism, from syphilis and sarcoma.

*Prognosis.*—The destructive processes of tuberculosis in bones and joints and the reparative processes which may follow are chronic in their course, extending over months or years, or sometimes alternating through a lifetime. It is pretty safe to assume that a child with bone or joint tuberculosis has other foci of the disease somewhere in its anatomy. There is more probability of the disease becoming general in a child, or becoming active in the meninges or elsewhere. But if it does not prove fatal through secondary lesions, there is more hope of repair of bone or joint than there would be in an adult.

A cured case of bone or joint tuberculosis may have a recurrence or one or the other foci may become active months or years after. Eradication by operation of a local lesion in bone or joint may not permanently cure the patient of tuberculosis; but it may prevent further absorption from that lesion. There is great danger of general infection resulting from operations upon tuberculous tissues. Septic infection of the tuberculous area adds greatly to the danger. Anyloid degeneration of the liver, spleen, kidneys, or intestinal villi are of very grave import. Individual cases differ so widely it is impossible to predict in the beginning what will be the final outcome of any given case. Relief from pain, reflex spasm, tenderness and swelling, with improved appetite and incrasing weight are all favorable indications.

*Treatment* of tubercular bone and joint diseases in general and local. The general treatment which is as important as the local measures in bone and joint tuberculosis, such as air and climate.

sunlight, exercise, rest and other hygienic measures, diet, drugs, tuberculin, etc., I will not touch upon in this paper. Rest is the most important of the agencies for local treatment, especially of diseases of joints, in bone diseases it does not give such evident good results, excepting in spinal cases.

Rest may be secured by lying in bed, by extension and counter-extension and by splints, which are means of fixation. Splints are of many materials and of many varieties. They are applied for fixation; that is, to prevent motion, and in some varieties also to produce traction. A curvious spine or even the whole skeleton may be splinted by a Bradford frame, or a wire gauze cuirass, or by plaster of Paris, or a steel brace; and the limbs by plaster or starch, or silicate of soda or steel, etc.

Traction is used by many surgeons for the purpose of pulling apart the inflamed joint surfaces and so relieving the irritation. It also relieves muscular tension and reflex spasm by tiring out the irritated muscles at the same time it does the work they were attempting to do—it prevents motion in the joint. Traction has a further use in correcting the faulty position of an extremity. It may be applied by a weight or by the weight of the limb itself, or by the tensions of a spring or other mechanical device. Fixation by splint has some effect to relieve these, but more slowly. Splints and traction in one or other form can either or both of them be used with the patient in bed or out of bed, as the case may require. Weight-bearing upon the lower extremity can be removed by raising the sound limb and using crutches, or by using a splint which takes its bearing at the pelvis. When bone or joint is secured at rest it should be in the most natural position for its usual function.

The only counter-irritant of marked power in joint tuberculosis is the actual cautery usually applied with the Paquelin apparatus. I have seen this used many times and I think it is very useful, especially if the joint is very painful.

Anaesthesia is generally necessary with children, though I have seen a boy of eight who preferred not to take the anaesthesia and bore the rapid application of the cautery bravely.

Injections of antiseptic or chemical substances have long been in use in tuberculosis of joints. A great many drugs and chemicals have been employed with the idea of destroying the bacteria or checking their development or of limiting their action upon the tissues or promoting repair of their ravages.

In my own experience the injection of iodoform emulsion has appeared more useful in cold abscess cavities than in joint tuberculosis, and is no longer used in joints.

At present formalin is in favor for injection in hydrops articuli; a two or four per cent. solution in glycerine. It should never produce tension.

Of late local hyperenia has come into some prominence as a therapeutic agent for tuberculosis of bones and joints. It is sometimes called the Bier treatment or the Bier-Klapp method, after Professor Bier of Born, and his assistant, Klapp.

If rest has been tried without avail, and injection and local hyperemine treatment have failed to check the disease, and in some cases which advance too rapidly to wait for these methods, there is no choice but to cut down upon the bone or joint and attempt an eradication of the disease. This is especially true if to the tubercular infection a pyogenic infection be added, or if a tubercualr infection has attacked the cpiphyseal line.

The whole tendency of practice of late years, in dealing with tubercular bone and joint disease is more conservative than formerly. The complete removal of all the diseased parts about the joint, including suppurating old rinuses and cavities, will sometimes avoid anyloid degeneration, prevent further dissemination of toxins and even of the tubercular disease itself. On the other hand, it is often very difficult to remove every particle of diseased tissue; and surgical trauma sometimes leads to a general dissemination of tubedculosis.

Resection should always be avoided if possible in children, or some form of atypical rather than typical resection be employed. It is often impossible to tell before the joint is opened whether the operation is going to be an arthrectomy or an atypical or typical resection.

When after any method of treatment the inflammation in a joint has subsided, considerable judgment is necessary in putting it to work again. Simple inflammatory disease may be dealt with more promptly and the patient given liberty in a week or two after the trouble has subsided. But with tubercular inflammation months rather than weeks must elapse before one can feel sure that the trouble will not return with use. There is much less danger of ankylosis in children's joints than in those of adults. And when, after operation, ankylosis is desired the bones should be held in position for months, sometimes for years, on account of this tendency to form a joint.

